# A Space Oddity: Geographic and Specific Modulation of Migration in *Eudyptes* Penguins

**DOI:** 10.1371/journal.pone.0071429

**Published:** 2013-08-02

**Authors:** Jean-Baptiste Thiebot, Yves Cherel, Robert J. M. Crawford, Azwianewi B. Makhado, Philip N. Trathan, David Pinaud, Charles-André Bost

**Affiliations:** 1 Centre d’Études Biologiques de Chizé, Unité Propre de Recherche 1934 du Centre National de la Recherche Scientifique, Villiers-en-bois, France; 2 Branch Oceans and Coasts, Department of Environmental Affairs, Cape Town, South Africa; 3 University of Cape Town, Animal Demography Unit, Rondebosch, South Africa; 4 British Antarctic Survey, Natural Environment Research Council, High Cross, Cambridge, United Kingdom; University of Tasmania, Australia

## Abstract

Post-breeding migration in land-based marine animals is thought to offset seasonal deterioration in foraging or other important environmental conditions at the breeding site. However the inter-breeding distribution of such animals may reflect not only their optimal habitat, but more subtle influences on an individual’s migration path, including such factors as the intrinsic influence of each locality’s paleoenvironment, thereby influencing animals’ wintering distribution. In this study we investigated the influence of the regional marine environment on the migration patterns of a poorly known, but important seabird group. We studied the inter-breeding migration patterns in three species of *Eudyptes* penguins (*E. chrysolophus*, *E. filholi* and *E. moseleyi*), the main marine prey consumers amongst the World’s seabirds. Using ultra-miniaturized logging devices (light-based geolocators) and satellite tags, we tracked 87 migrating individuals originating from 4 sites in the southern Indian Ocean (Marion, Crozet, Kerguelen and Amsterdam Islands) and modelled their wintering habitat using the MADIFA niche modelling technique. For each site, sympatric species followed a similar compass bearing during migration with consistent species-specific latitudinal shifts. Within each species, individuals breeding on different islands showed contrasting migration patterns but similar winter habitat preferences driven by sea-surface temperatures. Our results show that inter-breeding migration patterns in sibling penguin species depend primarily on the site of origin and secondly on the species. Such site-specific migration bearings, together with similar wintering habitat used by parapatrics, support the hypothesis that migration behaviour is affected by the intrinsic characteristics of each site. The paleo-oceanographic conditions (primarily, sea-surface temperatures) when the populations first colonized each of these sites may have been an important determinant of subsequent migration patterns. Based on previous chronological schemes of taxonomic radiation and geographical expansion of the genus *Eudyptes*, we propose a simple scenario to depict the chronological onset of contrasting migration patterns within this penguin group.

## Introduction

Migration is a widespread behaviour in the animal kingdom and is generally understood to be an adaptive mechanism in seasonal environments, by which individuals may compensate for locally unfavourable conditions outside the breeding period (review in [1], [2]). Migrating individuals may exploit other environments with supplementary gain (i.e., survival) compared with resident species. However, in the case of land-based marine species, migration after the breeding period may also reflect the release from breeding constraints, allowing inter-breeders to forage in more optimal habitats that may not be seasonal, but which are too distant for adults to use while raising their offspring on land (e.g. [3]). These scenarios prompt questions about what factors influence inter-breeding area location and hence migration direction in land-based marine species. Other factors known to promote the emergence of migration behaviour relate to memories of favourable sites and to the inherent historical factors individuals may carry [1], [4], [5]. Indeed, memories of profitable sites strongly decrease migration cost [1], [4], [6] and hence are likely to facilitate migration in animals such as seabirds that commonly exhibit high wintering-site philopatry [5], [7], [8]. By contrast, the role of historical influences on migration patterns has been little investigated in seabirds (but see [9]).

Seabird migration has mostly been considered for flying species [5], [10], [11], whereas migration movements of swimming/diving species have been little studied (but see [9], [12], [13]), mainly because of methodological issues [14]. Diving, flightless birds such as penguins are much more constrained in their large-scale movements than are volant seabirds because of their slower locomotion mode [6], so they may better integrate environmental modulation and reflect the influence of the site of origin on migration pathways. In the Southern Ocean, penguins represent nearly 90% of the avian biomass, consuming several million tons of marine resources annually, and they include both migratory and resident species [15]. Consequently, penguins are good candidates to provide a more general picture of migration strategies in marine organisms. Among extant penguin species, the crested penguins (genus *Eudyptes*) constitute the most diverse and abundant group. They are commonly found from the subtropics to Antarctica with some different species breeding in sympatry [15], [16]. During the inter-breeding period eudyptid penguins consistently migrate away from their breeding localities and remain at sea for half the year, with striking mechanisms of resource partitioning between neighbouring populations [8,17,18), as predicted from the ‘Hinterland’ model developed for at-sea distribution of breeding seabirds [19].

The main goal of the present study was to understand the extent to which the direction taken by migrating penguins to reach their wintering areas depends upon their site of origin, given the contrasting ages and past environmental influences of these sites, which are all in the same oceanic region. Our null hypothesis was that penguins from any species or site would all migrate in the same direction following the main marine currents governing the region, a major environmental factor that may influence penguins’ migration [9]. Travelling against the flow of oceanic currents is expected to be extremely costly for penguins, especially at the onset of their winter migration following a prolonged fasting period on land for moult [20], [21]. To reach our goal, we undertook tracking work at four sites in the southern Indian Ocean and followed the inter-breeding migration of three Eudyptes species, namely the macaroni *E. chrysolophus*, the eastern rockhopper *E. filholi* and the northern rockhopper *E. moseleyi* penguins, of which the two former are often found breeding in sympatry [16]. Based on our extensive tracking dataset, we made comparisons between sympatric species and between parapatric populations, examining: (1) the animals’ migration bearing towards their wintering area, with respect to the main currents governing the region, and (2) the inter-breeding marine habitat used. We assumed that birds had a strong evolutive inertia in both migration patterns and optimal habitats, based on previously published literature [1], [22].

Site-specific adaptations for each seabird population would facilitate partitioning of food resources while also leading to coherent at-sea distribution patterns among individuals from the same locality (e.g., [7]), while allowing for divergent patterns between different localities [8], [18], [23], [24]. This could be attributable to better food location and exploitation [25]–[27] and possible cultural effects at localities (e.g., [23]). Therefore, our first prediction was that inter-breeding migrating patterns depend more on the site than on the species for closely related species, reflecting these site-specific adaptations. Our second prediction was that despite these geographic adaptations, parapatric individuals would exploit a similar wintering habitat, in line with intrinsic life-history traits for the species (e.g., [28]). We attempted to match these site-specific migration patterns with the influence of the local paleoenvironment the penguins potentially experienced when they colonised the studied sites. To test our hypothesis about the relative effect of the site of origin versus that of the species on the penguins’ winter distribution, we used published plus novel datasets on penguins’ inter-breeding migration, and conducted niche modelling analyses.

## Materials and Methods

### Ethics Statements

All scientific procedures at the French Islands were approved by the ethics committee of the French Polar Institute (IPEV) and were conducted according to its guidelines and under permits of the Réserve Naturelle des Terres Australes Françaises and of the Comité de l’Environnement Polaire. On South-Africa’s Marion Island, a permit (# SE11-07) was granted by the South Africa’s Department of Environmental Affairs. The greatest care was taken to minimize stress while handling animals, which lasted less than 20 min in all cases.

### Study Sites and Species

**Figure 1:**
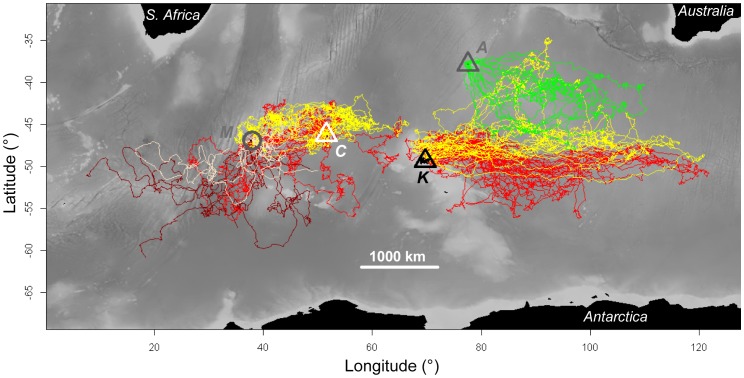
Interpolated tracks of *Eudyptes* penguins during their inter-breeding period in the Southern Indian Ocean. Depth contours are displayed in the background. Three species were tracked: macaroni *E. chrysolophus* (red and dark red lines), eastern rockhopper *E. filholi* (yellow and light yellow lines) and northern rockhopper *E. moseleyi* (green lines) penguins at four locations. Localities were: Marion (“M”, grey circle), Crozet (“C”, white triangle), Kerguelen (“K”, black triangle) and Amsterdam (“A”, grey triangle) islands. Penguins from Marion were tracked using satellite tags; on other localities (all symbolized by triangles), penguins were surveyed using GLS loggers.

The study took place in the southern Indian Ocean (Fig. 1), an oceanic region strongly influenced by the Antarctic Circumpolar Current (ACC), flowing eastwards. Circulation of the ACC in the western part of the study region is impacted by the warm southward flowing Agulhas Current [29]. Penguins were studied at four sites that together represent all the geological formations existing in the study region. From west to east these are: Marion, Crozet, Kerguelen and Amsterdam Islands, among which Marion and Amsterdam are the youngest in age, while Crozet and Kerguelen are much older (Table 1, [30]–[33]).

**Table 1:** Coordinates, environment and age of the four islands in the southern Indian Ocean from where the penguins were studied.

Island	Geographic coordinates	Oceanographic situation	Age (Ma)	*Eudyptes* species breeding
Marion	46°54′S, 37°44′E	Subantarctic	0.45	*E. chrysolophus*, *E. filholi*
Crozet (Possession Is.)	46°24′S, 51°45′E	Subantarctic	8.1	*E. chrysolophus*, *E. filholi*
Kerguelen	49°20′S, 69°20′E	Subantarctic	40	*E. chrysolophus*, *E. filholi*
Amsterdam	37°50′S, 77°31′E	Subtropical	0.40	*E. moseleyi*

The genus *Eudyptes* diverged from the other penguins about 15 Ma ago and in turn speciated within about the last 8 Ma in the New Zealand area [34]. Extant species are aged at approximately 3–8 Ma [34]–[36]. Today *Eudyptes* is the penguin genus with the highest species richness, with 8 extant species, despite the recent extinction of an eudyptid in New-Zealand, *Eudyptes chathamensis* [37]. These medium-size penguins are commonly found on Southern Ocean islands between 37°S and 62°S, where they breed annually in large colonies [16]. Three *Eudyptes* species were investigated in this study. We first focused on one of the largest eudyptids, the macaroni penguin *E. chrysolophus*, which is the greatest consumer of marine prey among all seabirds and the most numerous penguin [38], [39]. Secondly, we studied the smallest eudyptid, the rockhopper penguin, which was recently divided taxonomically into three species [40]. Two rockhopper penguins breed in the southern Indian Ocean, namely the eastern species *E. filholi*, a common subantarctic penguin, and the northern species *E. moseleyi*, which is restricted to the subtropics. We studied *E. chrysolophus* and *E. filholi* at the subantarctic Marion, Crozet and Kerguelen islands, where they breed sympatrically but with a 3-week difference in their breeding phenology [41]. In contrast, *E. moseleyi* has an earlier and longer breeding cycle [42], and was studied at subtropical Amsterdam Island (see details on migration schedule at each locality in Table S1 and [8], [13], [18], [43], [44]).

### Tracking Techniques

Penguins were instrumented with one of the two following tracking devices when moult was complete on land, *i.e.*, before the birds’ departure for migration during the inter-breeding period at sea (Table 2). Animals from Marion (n = 24) were equipped with ARGOS Platform Terminal Transmitters (PTTs) that emit signals to satellites allowing the calculation of their position [45]. These PTTs were fitted medially to the lower back to reduce drag, and fixed to the back feathers using cyanoacrylate glue (Loctite 401) and plastic cable ties. Devices used in 2005, 2006 and 2007 measured 91*48*21 mm (45 g); and in 2008 90*34*24 mm (30 g). They were duty-cycled to transmit for 8 hours with a transmission rate of 60 s and to switch off for the next 16 hours. Penguins from Crozet (n = 40), Kerguelen (n = 57) and Amsterdam (n = 20) were equipped with miniaturized light-based geolocation positioning devices (GLSs, British Antarctic Survey, Cambridge, UK). These devices were leg-mounted using specially designed flexible leg bands, following [13]. GLS loggers record ambient light level and time, allowing the estimation of latitude and longitude twice a day [46], [47]. GLS tags also recorded ambient sea temperature, once during every 20 min period of continuous immersion, with a resolution of 0.0625°C and an accuracy of ±0.5°C. After the GLSs were recovered, logged data were analyzed following previously published methods [48], using the package ‘tripEstimation’ in R 2.9.0 [49] and assuming a mean daily travelling speed of 2 km•h^−1^ [50] in order to estimate the most probable track. Location estimates in this case are not as accurate as for PTTs (tens to hundreds kms versus <1 km in the best cases, respectively [51], [52]), and GLSs need to be recovered in order to collect the data, unlike in the case of satellite linked PTTs. However, the larger satellite tags with their antennae are more likely to produce adverse effects such as additional hydrodynamic drag on the foraging efficiency of these streamlined diving birds, especially over prolonged periods [14], [53]. The total number of animals instrumented amounted to 141, with most of these (104 individuals) tracked during the same year (2007) from the four sites. Detailed information about the winter habitat used by penguins from Crozet, Kerguelen and Amsterdam Islands is provided for each species in published papers [8], [13], [18].

**Table 2:** Summary of tracking devices used to study inter-breeding movements of *Eudyptes chrysolophus*, *E. filholi* and *E. moseleyi* penguins.

Species tracked	Locality	Year	Animals instrumentedn (♂–♀)	Colony atlocality	Device used (weight)
*E. chrysolophus*	Marion	2005	2 (1–1)	Macaroni Bay North	PTT – Telonics ST-10 (45 g)
*E. chrysolophus*	Marion	2007	6 (4–2)	Swartkop, Kildalkey,Bullard North	PTT – Telonics ST-10 (45 g)
*E. chrysolophus*	Marion	2008	6 (3–3)	Swartkop, Bullard North	PTT – Sirtrack Kiwisat 202 (30 g)
*E. chrysolophus*	Crozet	2007	18 (9–9)	Jardin Japonais	GLS - BAS MK4 (6 g)
*E. chrysolophus*	Kerguelen	2006	21 (11–10)	Cap Cotter	GLS - BAS MK4 (6 g)
*E. chrysolophus*	Kerguelen	2007	16 (8–8)	Cap Cotter	GLS - BAS MK4 (6 g)
*E. filholi*	Marion	2006	2 (1–1)	Trypot	PTT – Telonics ST-10 (45 g)
*E. filholi*	Marion	2007	2 (?–?)	Trypot	PTT – Telonics ST-10 (45 g)
*E. filholi*	Marion	2008	6 (?–?)	van den Boogaard, Swartkop	PTT – Sirtrack Kiwisat 202 (30 g)
*E. filholi*	Crozet	2007	22 (11–11)	Pointe Basse	GLS - BAS MK4 (6 g)
*E. filholi*	Kerguelen	2007	20 (10–10)	Île Mayes	GLS - BAS MK4 (6 g)
*E. moseleyi*	Amsterdam	2007	20 (14–6)	Entrecasteaux	GLS - BAS MK4 (6 g)

### Analytic Tools Used

For all analyses we used R 2.9.0 [49]. Unreliable Argos locations were removed using the algorithm from the ‘argosfilter’ R package [54], with an upper-threshold speed of 2.1 m s^−1^ according to previous measurements [55]. In order to standardize the frequency of locations available along the tracks, we re-sampled the tracks obtained and made linear interpolations to conform to the 12 h frequency of GLS-derived estimates, using R packages ‘sp’ and ‘trip’. Locations received from the PTTs were thereafter analysed in the same way as GLSs to standardise interpretation of all the tracks.

Bearing was calculated between the point of origin at the colony and the farthest point reached for each animal studied, using ‘circstat’ package. This was expressed as a circular measurement in degrees, with 0° equivalent to a northwards direction. We used circular analysis of variance with ‘high concentration F-test’ in R package ‘circular’ to compare bearings between sites or species. We excluded from these analyses the shortest tracks from Marion Island (duration <15 d: 1 *E. chrysolophus* and 2 *E. filholi*) that were probably caused by early battery failure. As a consequence, we assumed that bearings inferred from tracks over 15 d indicated the directions of wintering destination of the penguins, which seems to be the case in these species, which typically migrate directly towards population-specific wintering areas [8], [13], [18].

As GLS-derived location estimates are less precise than PTTs to depict wintering destination of the penguins, we also analyzed monthly average temperature records to compare seawater temperature used, possibly reflecting a latitudinal shift, between species. We carried out Student’s *t*-tests to compare these monthly-averaged temperature records between species. For the three sites where two species of *Eudyptes* penguin breed sympatrically, all locations available for the inter-breeding period of each species were also binned by degree of latitude. From this dataset, Student’s *t*-test was again used to examine for statistical differences in the latitudinal distributions of species. In all tests the threshold for significant differences was set at p = 0.05.

Habitat suitability for the penguins during the wintering period (as defined below) was modelled using Mahalanobis Distances Factor Analysis (MADIFA, [56]) in R package ‘adehabitat’. This method is appropriate for building habitat suitability maps from presence-only data, such as tracking data (for a comparison of methods see [57]). In the MADIFA, two principal components analyses (PCAs) successively summarize available information comprising: (a) the environment described by spatial variables; and (b) the relationship between the locations of animals and the environment. Environmental variables used were bathymetry (BATHY) and its gradient (BATHYG), sea-surface temperature (SST) and its gradient (SSTG), SST anomalies (SSTA), sea-surface chlorophyll a concentration (CHLA), mixed-layer depth (MLD) and eddy kinetic energy (EKE). MLD was a mean of annual data obtained since 1941. Previous studies have shown that these variables can be used to model at-sea movements of penguins (see [58]–[60]). The temporal resolution selected for dynamic variables was one month, and the spatial grid 1° in accordance with the geolocation technique accuracy. The spatial data were obtained from the NOAA’s ETOPO (http://www.ngdc.noaa.gov/mgg/gdas/gd_designagrid.html?dbase=GRDET2), the Bloomwatch 180 (http://coastwatch.pfel.noaa.gov/coastwatch/CWBrowserWW180.jsp), the LOCEAN (http://www.locean-ipsl.upmc.fr/~cdblod/mld.html) and the AVISO (http://las.aviso.oceanobs.com/las/servlets/dataset) websites. We modelled winter at-sea distribution of the two species that were studied at more than one site (that is, *E. chrysolophus* and *E. filholi*). We focused on the year 2007 when most of the tracking data were collected and all sites were sampled. The habitat model was based on the at-sea distribution of the birds from Crozet, and the model predictions were projected on the whole study area in order to compare predictions with the actual locations of the birds from all sites. We chose Crozet as a reference site for habitat modelling since it has an intermediate longitudinal location between the two other sites. The time window for modelling wintering habitat was one month, according to seasonality in this oceanic region [61], and taking into account the minimum mobility of the birds (that suggests intensive use of a wintering area, see [13]), which occurred in July for *E. chrysolophus* [8], [13], September for *E. filholi* and May for *E. moseleyi* [18].

## Results

From the 141 animals instrumented in the four sites we obtained 87 tracks, with 62 from the 2007 inter-breeding season.

### Satellite-tracking from Marion Island

PTTs transmitted locations for 11 *E. chrysolophus* individuals from Marion, over periods from 14.7 d to more than 205 d (mean±SD: 90.6±73.5 d). Among these, devices used in 2008 transmitted considerably longer (171.5±32.4 d). For *E. filholi*, 10 animals were followed, from 4.9 to 120.8 d (60.9±45.9 d all years pooled, and 99.8±20.6 in 2008). One PTT was recovered from *E. chrysolophus* in spring 2008.

### Archival Tags from Crozet, Kerguelen and Amsterdam Islands

For GLS-equipped animals, 36 *E. chrysolophus* (65.5%) and 26 *E. filholi* (62%) were recaptured on Crozet and Kerguelen Islands, and 14 *E. moseleyi* (70%) on Amsterdam Island. Data which could be downloaded comprised 30 GLSs from *E. chrysolophus*, 25 from *E. filholi* and 11 from *E. moseleyi*.

### General Inter-breeding Migration Patterns for the Study Birds

**Figure 2:**
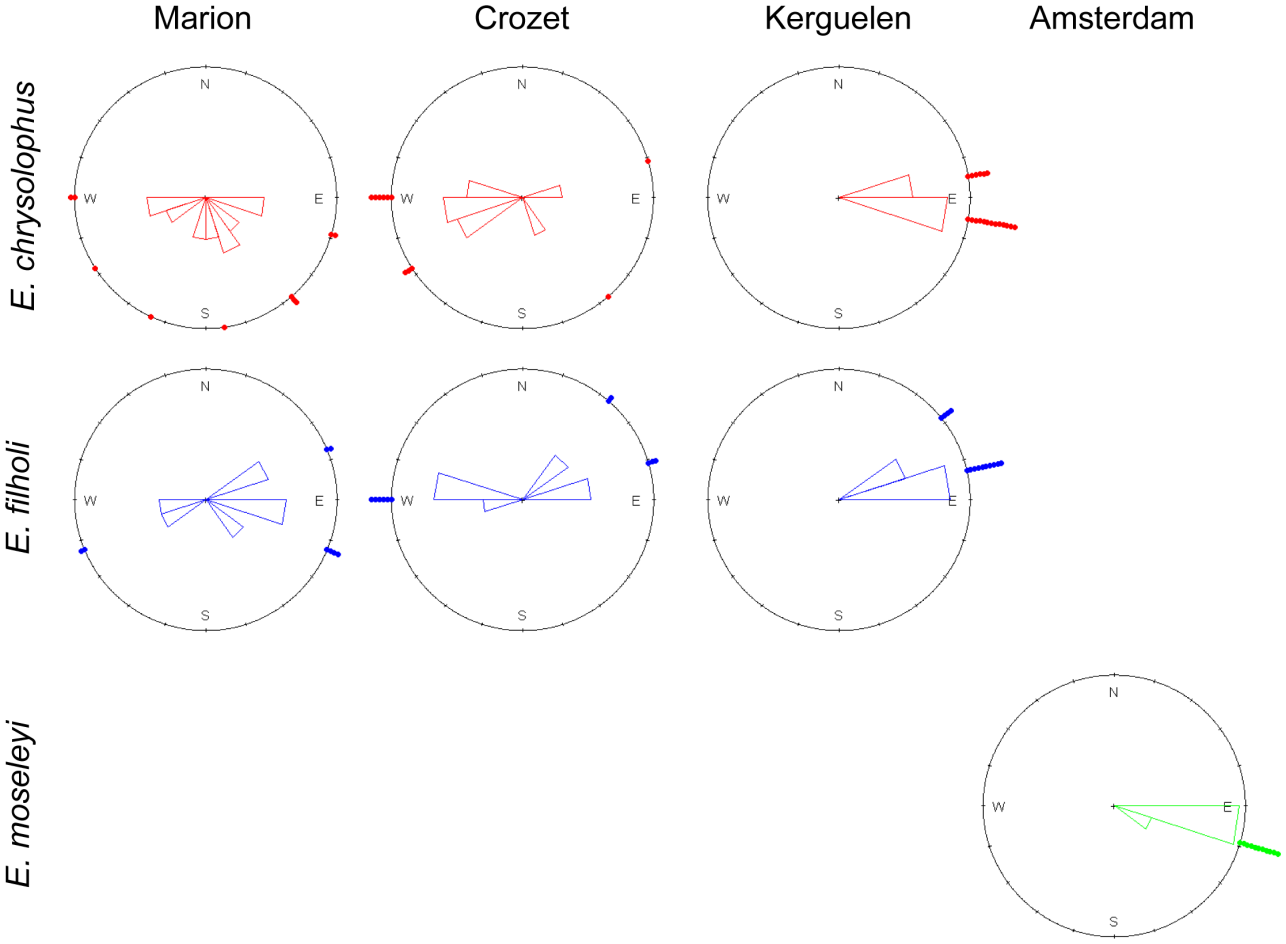
Outbound migration bearings of each sampled *Eudyptes* population. Geographical direction of the farthest point reached from the colony for all individuals tracked was used to determine bearing.

Tracked *Eudyptes* penguins performed long-range inter-breeding movements (Fig. 1), travelling thousands of km. These penguins concentrated in two areas: firstly to the west of Crozet, comprising penguins of the western sector (i.e. from Crozet and Marion), and secondly east of Kerguelen, with penguins from Kerguelen and Amsterdam. All penguins remained in the study region for the complete inter-breeding period, except a few individuals from Marion that reached the southern Atlantic Ocean (at least three *E. chrysolophus* and one *E. filholi*, with maximum ranges of 1993, 2239, 1772 and 1588 km, respectively). Penguins from Marion, and to a lesser extent from Crozet, showed higher angular variance in bearing (0.84 and 1.06 versus 0.62 and 0.77 for *E. chrysolophus* and *E. filholi*, respectively) than those from Kerguelen and Amsterdam, which typically migrated in a very narrow range of directions (0.01, 0.04 and 0.01 for *E. chrysolophus*, *E. filholi* and *E. moseleyi*, respectively, Fig. 2). When pooled together by site, *Eudyptes* penguins at each site had significantly different average bearings to those from all other sites (Table 3).

**Table 3:** Statistical comparison of migration bearings for *Eudyptes* penguins from their respective breeding sites.

Localities compared (no. individuals)	Circular Analysis of Variance
Marion (18)/Crozet (22)	F_1_ = 24.8, p<0.01
Marion (18)/Kerguelen (33)	F_1_ = 18.7, p<0.01
Marion (18)/Amsterdam (11)	F_1_ = 5.3, p = 0.03
Crozet (22)/Kerguelen (33)	F_1_ = 89.6, p<0.01
Crozet (22)/Amsterdam (11)	F_1_ = 106.7, p<0.01
Kerguelen (33)/Amsterdam (11)	F_1_ = 13.8, p<0.01

Maximum distances from breeding localities were used to determine bearings. The number of individuals compared is indicated in brackets.

### Comparisons between Sympatric Species

**Figure 3:**
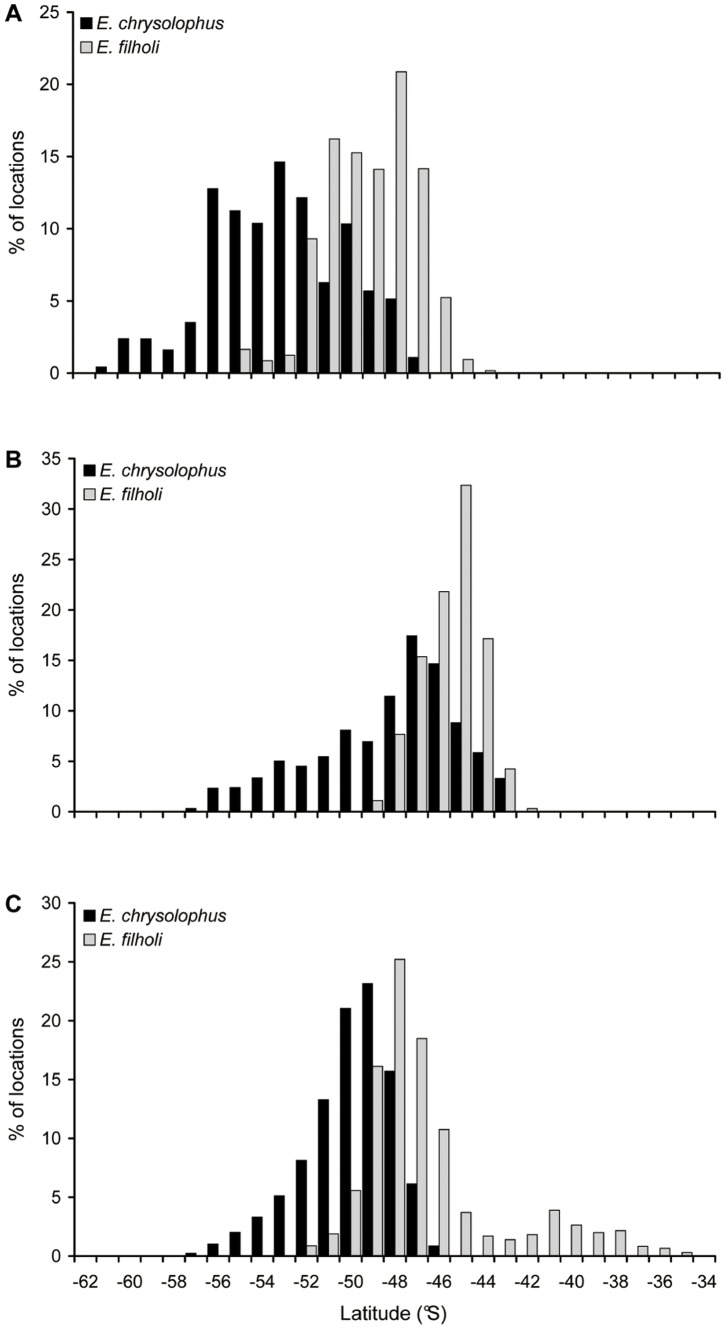
Latitudinal distributions of the two sympatric *Eudyptes* species. Penguins from (A) Marion, (B) Crozet and (C) Kerguelen Islands.

**Figure 4:**
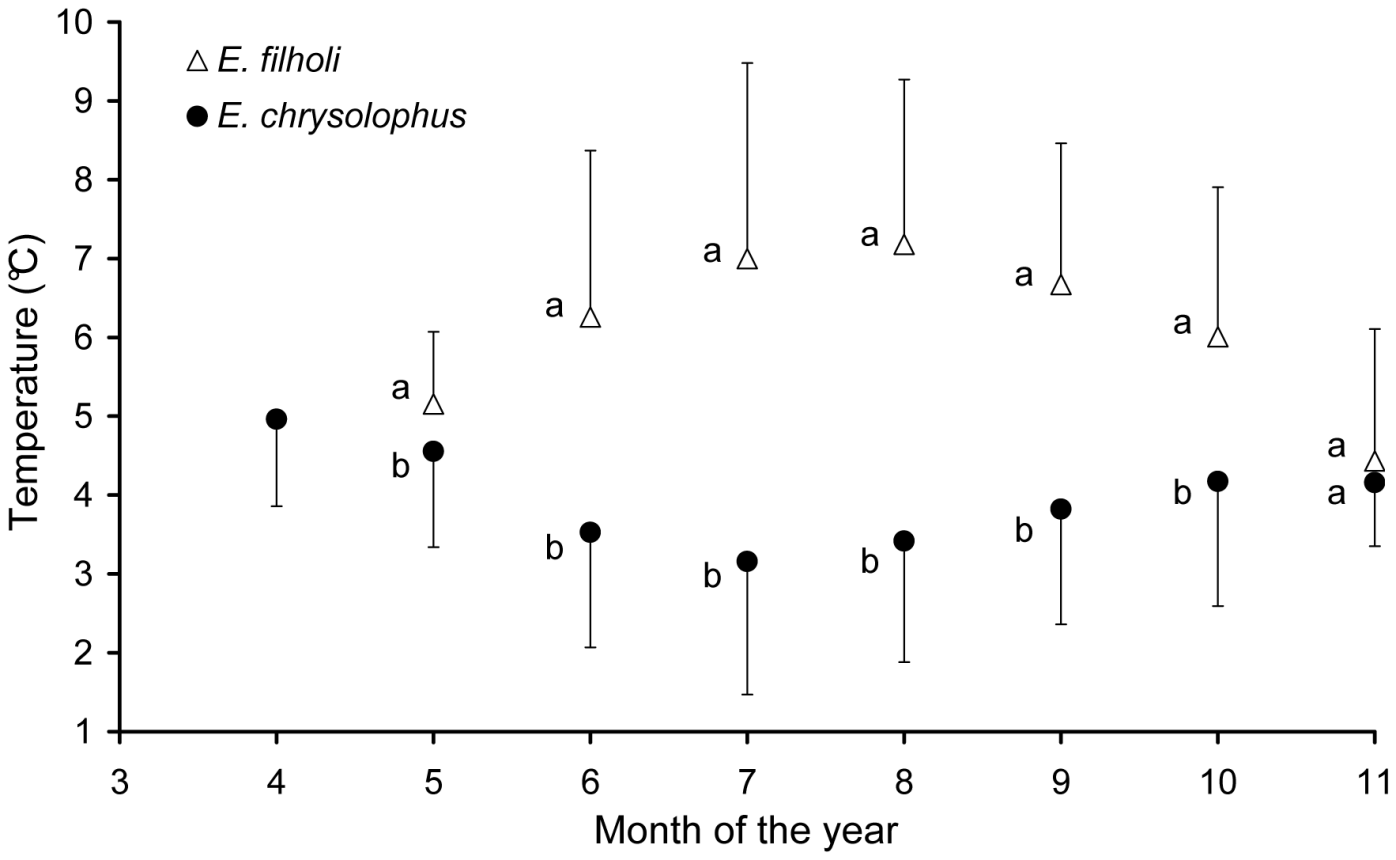
Mean temperature recorded by the GLS devices fitted on penguins from Crozet and Kerguelen. Values are mean+SD for *E. filholi* and mean - SD for *E. chrysolophus*. Different letters indicate significantly different (p<0.05) monthly means between the two species; for letters that are the same there was no significant difference.

Bearings at maximum range were not significantly different between sympatric species, for all three sites studied with more than one species (Fig. 2, Table 4). For each site where they occurred together, *E. chrysolophus* dispersed significantly more southerly than *E. filholi* (t_6630_ = −65.7, t_5445_ = −51.1, t_8119_ = −69.5 for Marion, Crozet and Kerguelen, respectively, all p<0.00001, Fig. 3). This was confirmed by the ambient sea temperature records from the GLSs of the animals from Crozet and Kerguelen, with *E. chrysolophus* distributing in colder waters than *E. filholi*, except during the end of their at-sea period, when birds of both species were distributed close to their breeding localities (Fig. 4).

**Table 4:** Statistical comparison of migration bearings for sympatric species of *Eudyptes* penguins from their respective breeding sites.

Locality	*E. chrysolophus/E. filholi* (no. individuals)	Circular Analysis of Variance
Marion	(10)/(8)	F_1_ = 2.2, p = 0.16
Crozet	(11)/(11)	F_1_ = 2.7, p = 0.12
Kerguelen	(19)/(14)	F_1_ = 3.6, p = 0.07

The number of individuals compared is indicated in brackets.

### Comparisons between Parapatric Populations

In both *E. chrysolophus* and *E. filholi*, outbound migration bearings were significantly different between penguins from one site to any other one (Table 5).

**Table 5:** Statistics comparison of migration bearings between parapatric populations of *Eudyptes* penguins.

Species	Localities compared (no. individuals)	Circular Analysis of Variance
*E. chrysolophus*	Marion (10)/Crozet (11)	F_1_ = 9.4, p<0.01
*E. chrysolophus*	Marion (10)/Kerguelen (19)	F_1_ = 29.9, p<0.01
*E. chrysolophus*	Crozet (11)/Kerguelen (19)	F_1_ = 162.2, p<0.01
*E. filholi*	Marion (8)/Crozet (11)	F_1_ = 12.5, p<0.01
*E. filholi*	Marion (8)/Kerguelen (14)	F_1_ = 6.8, p = 0.02
*E. filholi*	Crozet (11)/Kerguelen (14)	F_1_ = 11.3, p<0.01

Maximum distances from sites were used to determine bearings. The number of individuals compared is indicated in brackets.

#### Eudyptes chrysolophus

**Figure 5:**
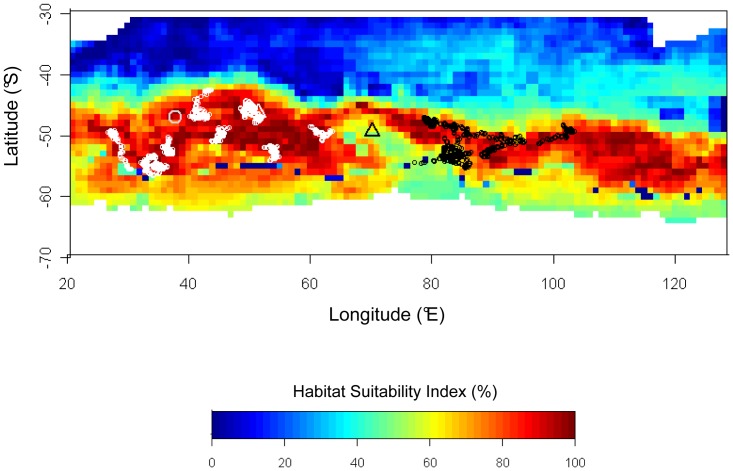
Outputs of MADIFA habitat suitability modelling for *E. chrysolophus*. Map of winter habitat suitability predicted, with observed winter distribution of conspecifics. The model was based on the distribution of animals from Crozet only, during the month with minimum mobility (July). Locations of the colonies are indicated: Marion (grey circle), Crozet (white triangle) and Kerguelen (black triangle). Locations of the animals from Crozet (white) and Kerguelen (black) during the corresponding month are shown; no data available from Marion in July 2007.

Winter habitat modelling of *E. chrysolophus* from Crozet, based on location data from July 2007, showed the primary importance of SST on the first axis of the first PCA and of BATHYG on the second axis (Table S2). The second PCA showed the highest scores for SST and SSTG on the first axis, which dominated variance explanation. The projection of this habitat suitability model showed a band of maximum suitability level between 45 and 55°S (dark red, Fig. 5): this band was wider in the Marion-Crozet region and east of Kerguelen (100–120°E), while interrupted west of 25°E and in the vicinity of Kerguelen. The locations of the wintering *E. chrysolophus* from Crozet during July 2007 logically matched high levels of suitability (92.2±7.6%, Fig. 5) and importantly so did those from Kerguelen (74.2±16.9%), albeit some locations fell south of the areas predicted as the most suitable (50.0±28.6%). No *E. chrysolophus* locations were available in July 2007 from Marion.

#### Eudyptes filholi

**Figure 6:**
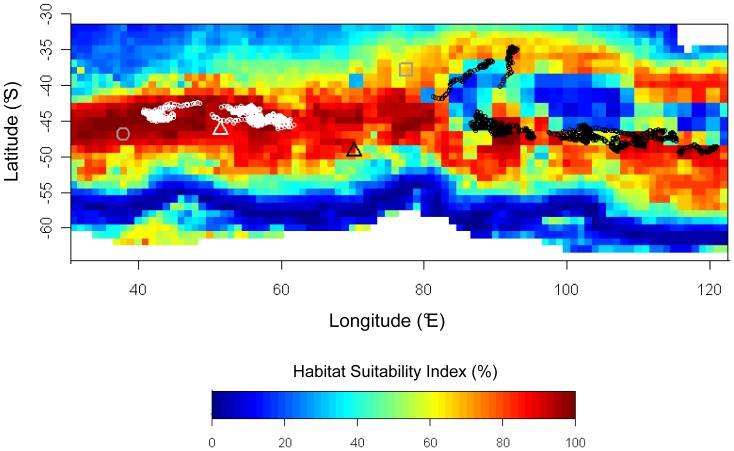
Outputs of MADIFA habitat suitability modelling for *E. filholi*. Map of suitable winter habitat predicted, with observed winter distribution of conspecifics. The model was based on the distribution of animals from Crozet only, during the month with minimum mobility (September). Locations of the colonies are indicated: Marion (grey circle), Crozet (white triangle), Kerguelen (black triangle) and Amsterdam (grey square). Locations of the animals from Crozet (white) and Kerguelen (black) during the corresponding month are shown; no data are available from Marion for September 2007.

Habitat modelling for *E. filholi* from Crozet during September 2007 showed the importance of CHLA and SST on the first axis of the first PCA, but also of BATHY and BATHYG on the second axis (Table S3). On the second PCA, variance was almost entirely captured on the first principal component, revealing the primary influence of SSTG on the winter distribution of *E. filholi*. Mapping of habitat suitability showed in this case a latitudinal band of more suitable habitat around 45°S, that separated into two branches east of 80°E (Fig. 6). Between these two branches occurred very low levels of suitability (0–20%), where the deepest values of MLD were found in the study area. The locations of *E. filholi* from Crozet in winter matched high suitability levels (97.9±2.1%) just north of Crozet, while for the Kerguelen birds, locations fell along the edges of the expected suitable habitat (66.2±25.6%). However, Kerguelen birds closely followed the dichotomic pattern predicted for habitat suitability (Fig. 6). No data from Marion were available for September 2007.

## Discussion

Our investigation generates new insights into the inter-breeding period and winter biology of *Eudyptes* penguins at both species and population levels [13], [17], [62]. First, eudyptids (all species pooled) showed site-specific migration bearings. Second, at each site similar compass bearings were observed between sympatric species, though *E. chrysolophus* was consistently distributed in colder waters than *E. filholi*. And third, within each species we found different migration patterns for populations from different sites, although individuals foraged in similar environments. These results show that inter-breeding migration patterns in a group of sibling seabird species depend primarily on the site of origin and secondly on the species. Such site-specific migration bearings, together with similar wintering habitat used by parapatrics, support the hypothesis that migration behaviour is affected by the intrinsic characteristics of the originating site [63]. In this study two kinds of positioning devices were used to track penguin migration: Argos PTTs and GLS loggers, with the former providing better spatial accuracy (see Methods section). However, compared to the ocean-wide scale of our study the different instruments used will not impact our conclusions, especially since we accounted for the low accuracy of GLSs in the habitat modelling resolution.

### Main Environmental Factors Driving the *Eudyptes* Distribution

The MADIFA approach showed the general importance of SST, SSTG, BATHYG and CHLA as the main environmental factors affecting *Eudyptes* penguin distributions during the inter-breeding period. High levels of MLD appeared negatively to affect habitat suitability for *E. filholi*: birds from Kerguelen were distributed at the periphery of the area where the highest levels of MLD (over 200 m) were found. For both species, predictive maps produced for the habitat used by individuals from Crozet corresponded well with observed distribution patterns of animals from Kerguelen. For both species also, the model predicted suitable habitat at more southerly latitudes in the Marion region than in the Crozet region, which is consistent with water mass circulation in this sector [29]. Finally, *E. chrysolophus* tracked from Marion in 2008 appeared to distribute according to the model predictions (see Fig. 1 and Fig. 6), though the model was based on 2007 data. These results add support to the notion of low inter-annual variability in winter feeding grounds for eudyptid penguins [8] and hence the validity of our habitat modelling approach [3]. At a finer scale, penguins from Marion exhibited the largest variance in migration bearing, but we could not test for a potential effect of the colony of origin at this island because too few individuals were sampled from each colony (Table 2). However the small size of the island (area: 290 km^2^) argues against this potential effect because at the much larger Kerguelen Island (area: 7215 km^2^), the different species tracked from distinct colonies showed similar bearings. Finally, we recall that rockhopper penguins from Amsterdam Island (*E. moseleyi*) are now considered to belong to a separate species than *E. filholi* [40], which precluded including the former in the habitat suitability modelling of the latter. In any case, it would have been necessary to carry out such analyses separately for penguins from Amsterdam owing to the time shift in their migration schedule compared to rockhopper penguins from the other sites.

### Population-based Strategies: Evolutionary Implications

Our large-scale study shows clear site-specific migratory patterns among the 4 islands. The fact that 96% of seabirds breed in colonies probably favours emergence of such site-specific migration patterns in these organisms: the possibility of individuals communicating and sharing information within the colony has been debated for a long time [25]–[27]. The existence of such strategies in our study reveals a major selective advantage to migrate to and exploit certain marine areas according to an animal’s origin, thereby maximizing winter food gains at an individual scale. Synchronized departure and return in eudyptids, together with highly coherent at-sea distribution, at-sea observations of flocks of individuals and possible synchronized dives between individuals [8], [18], [64], [65] all suggest that penguins are strongly influenced by group dynamics in their foraging strategies in general. Such characteristics favour indeed the emergence of population-based foraging strategies [66]. Further, this site-specific migration behaviour suggests that the spatial heterogeneity of favourable habitats in the southern Indian Ocean is not recent, and may be significant in shaping penguin populations’ evolution (and possibly population trend, [17]). Recently, segregation of populations outside the breeding period has been identified as a strong barrier to gene flow in seabirds, and especially in penguins [67]. These behavioural mechanisms thus potentially drive genetic divergence in *Eudyptes* populations, with implications for sub-speciation and eventually speciation through reproductive isolation [68].

### Species Segregation in Winter

At the species level, winter tracking showed that macaroni penguins consistently wintered in colder, more southerly waters (the ‘Polar Frontal Zone’, see [8]) than did the sympatric rockhopper penguins (the ‘Subantarctic Zone’, see [18]), thus confirming previous inferences from dietary stable isotopes analyses [62]. Hence, spatial segregation is the main mechanism involved in resource partitioning between these closely-related species. Previous studies conducted during the breeding period showed only partial if any segregation of sympatric eudyptids on every ecological axis investigated: breeding chronology [41], foraging range and habitat [55], [69], diving behaviour [70] and diet [71], [72]. However, it has often been emphasised that sympatry in eudyptids involves no more than two species that include the smallest (the rockhoppers), in low numbers, together with one of the largest species (Macaroni, Royal *E. schlegeli* or Erect-crested *E. sclateri* penguins) [16]. Knowing the importance of size and body mass on penguins’ diving behaviour [73], this suggests that co-existence is probably also related to the vertical component of the birds’ foraging behaviour. Therefore, we can assume that during the breeding season, when sympatric penguins are more constrained to return frequently to their colonies, and thus cannot segregate at a large spatial scale, their respective niches may be separated by the conjunction of all partial segregating mechanisms in time, space (horizontal and vertical components) and trophic resources, as it is the case in other congeneric penguins [74]. Outside the breeding period, the situation seems more straightforward, since the birds may distribute on a larger scale at that time without returning to the colonies and thus display clear-cut spatial segregation. Further, the small delay in the migration schedule may even be viewed as an adaptive mechanism allowing a decrease in inter-specific competition for food [55] by decreasing the at-sea overlap between both species during the departure period, when birds have poor body condition after their moulting fast [20], [21].

### Evolutionary Inertia of the Migration Program

The locations of the breeding grounds and of suitable winter feeding habitat must have an important influence on migration bearings. However, in mammals, some populations migrate to a specific geographic destination even though the targeted habitat may have been strongly altered [75], suggesting that there may be elements of evolutionary inertia in the inherited migration program [1]. For some birds, expanding populations may have retained their original, but modified or apparently sub-optimal, winter quarters and migration routes [22]. Interestingly, all such cases have been reported for species whose juveniles migrate independently from the adults [76], [77]. This evolutionary inertia suggests that migration patterns that are observed at a given time are not necessarily optimal at an evolutionary time scale and supports the hypothesis of a strong influence of paleoenvironments on site-specific migration patterns. In most seabirds, including penguins, emancipation of juveniles is generally not synchronous with the post-breeding migration of adults [15], [78]. Thus, inter-generational learning may be limited in these animals and evolutionary inertia for migration programmes would be strong in adults, an idea supported by the strong inter-annual fidelity observed in their wintering areas [5], [7], [8]. Moreover, eudyptid penguins are associated with well-defined habitats during the inter-breeding period, notably regarding SST as revealed by our study and delimited by oceanographic fronts [8], [18]. It is probable that large-scale shift of these boundaries over geological time scales towards or away from a breeding location, and the resulting changes in food available within the swimming range of penguins [16], have had an influence on their inter-breeding migration patterns. This inertia may explain why in our study the penguins from Crozet appear to behave paradoxically in the current situation. Indeed, the vast majority of these eudyptids from Crozet swam against the main flow of the ACC at the onset of their winter migration, while (1) such movements are expected to be costly, particularly after the prolonged fasting period spent on land during moult [20], [21], and (2) suitable habitats must be available for both species at only moderate distances eastwards (Figs. 5 and 6). In contrast, other penguin species have been shown to have migration facilitated by currents [9].

### A Scenario for Site-specific Onset of *Eudyptes* Migration

Here we propose a simple, plausible scenario based on previous work on taxonomic radiation [34], [35] and molecular biogeography [36] of the genus *Eudyptes*, that may explain the site-specific migrating schemes observed in our study. We recall here that a fundamental assumption is that *Eudyptes* penguins’ current ecological optimum in terms of winter habitat remains similar over the entire period considered (see [8], [18], this study). Since our results pointed out the influence of SST on penguins’ habitat, this scenario also integrates the historical latitudinal movements of the water masses in the southern Indian Ocean that have been depicted from analysis of sediments in the Southern Ocean seafloor [79], [80].

In the southern Indian Ocean the first sites which could have been colonised by *Eudyptes* penguins were probably Kerguelen and Crozet, the oldest ones. This colonisation may have taken place as early as 5 Ma ago (separation of the clades “macaroni” and “rockhopper”), but more probably later, owing to subsequent speciation within this group (3 Ma ago, [35]) with geographic range extension around the Southern Ocean along the ACC pathway and away from their New-Zealand origin. It is likely that Kerguelen penguins developed an inter-breeding migration strategy directed with the main flow of the ACC (i.e., eastwards), due to the lower energetic cost of this strategy (Fig. S1A). However, Crozet penguins would have developed an opposite strategy, because in the early Pleistocene (from 1.9–1.3 to 0.9–0.42 Ma ago) there was a prolonged period of intense cooling [79], [80] that may have driven penguins from Crozet to migrate towards the northwest to reach the closest warmer, optimal wintering waters advected by the Agulhas Current. This cooling period lead surface isotherms to be located at more northerly latitudes (by nearly 10°) than those occupied today [80]. At that time, Kerguelen penguins likely also adjusted the latitudinal component of their inter-breeding migration but keeping their eastwards longitudinal component (Fig. S1B). Then, from 0.9–0.42 Ma ago, climate warmed during the mid-Pleistocene transition and caused the Southern Ocean water masses to shift southwards. Owing to the importance of SST to these species’ habitat suitability, we assume penguins would have modified their migration routes in response to this phenomenon. More recently (0.45–0.40 Ma ago), Marion and Amsterdam Islands emerged: Marion centred on the eudyptid’s wintering habitat and Amsterdam to the north of it. Therefore, *Eudyptes* penguins that colonized Amsterdam from subantarctic islands [36] would have developed a migration route directed south-eastwards, accounting both for optimality to reach more southerly habitats, and to travel with the main flow of the ACC (Fig. S1C). Penguins colonizing Marion Island would have been less constrained in the direction towards which they migrate, because of the location of this island in the favourable habitat exploited by the penguins.

### Conclusions and Perspectives

Our study suggests an influence of paleoenvironments in the different inter-breeding migration patterns for populations of seabirds such as penguins. To our knowledge, only one other study [63] attributed the divergent winter migration patterns observed in penguins to such possible historical influences. Our putative scenario is probably much simplified compared with the successive environmental events and other ecological factors, which all have led to the different strategies that are currently observed. Nevertheless, our proposed scheme explains how these strategies may be more site-specific than species-specific for this homogenous penguin group. Importantly, this scenario supports the hypothesis that the longitudinal component of large-scale movements seems to be a deep, site-specific life-history trait, as it is shaped by the paleoenvironmental conditions governing the site. Conversely, the latitudinal component seems much more variable as populations would be able to finely adjust this component given local variation in the environment. However, limits to this plasticity may be reached in case of rapid changes in the environment, as seems to be the case today [60], [81].

Our study also emphasizes the benefit of the comparative approach in tracking survey analyses. Comparison of winter migration patterns from multiple sites (e.g., [82]) and/or species (e.g., [10], this study) provides an understanding of ocean-scale movements of animals that is invaluable for conservation purposes. In our study, *E. moseleyi* was the species suffering the worst conservation status (listed as ‘endangered’, [83], [84]). Yet, it was also the only species in our study for which we could not compare parapatrics. In order to investigate fidelity in its environmental niche and promote conservation of this threatened species, it is urgently needed to track birds from the Tristan da Cunha group in the southern Atlantic Ocean, the only other region where it is distributed.

## Supporting Information

Figure S1 Illustration of the chronological scenario proposed from the paleoenvironments to explain the *Eudyptes* penguins’ contrasted inter-breeding migration patterns. Cool period during early Pleistocene (A, from 1.9–1.3 to 0.9–0.42 Ma ago), with penguins at Crozet and Kerguelen Islands and putative migration routes (yellow arrows); then (B) warming during the mid-Pleistocene transition (from 0.9–0.42 Ma ago); and (C) emergence of Marion and Amsterdam Islands from 0.45–0.40 Ma ago, with putative migration routes for penguins from these islands (white arrows). Shaded areas symbolize supposedly suitable winter habitat for each period. Warm Agulhas (orange arrow) and cool Antarctic Circumpolar (blue arrow) currents are also indicated. (TIF)

Table S1 Migration schedule (peak departure/return dates from the colony) and tagging period of the three species surveyed (the macaroni *Eudyptes chrysolophus*, the eastern *E. filholi* and the northern *E. moseleyi* rockhopper penguins) on the four localities (Marion, Crozet, Kerguelen and Amsterdam Islands). References: *. this study, 8. Thiebot JB, Cherel Y, Trathan PN, Bost CA (2011) Inter-population segregation in the wintering areas of macaroni penguins. Mar Ecol Prog Ser 421∶279–290. 13. Bost CA, Thiebot JB, Pinaud D, Cherel Y, Trathan PN (2009) Where do penguins go during the interbreeding period? Using geolocation to track the winter dispersion of the macaroni penguin. Biol Lett 5∶473–476. 18. Thiebot JB, Cherel Y, Trathan PN, Bost CA (2012) Coexistence of oceanic predators on wintering areas explained by population-scale foraging segregation in space or time. Ecology 93∶122–130. 43. Crawford RJM, Cooper J, Dyer BM (2003) Population of the Macaroni Penguin *Eudyptes chrysolophus* at Marion Island, 1994/95–2002/03, with Information on Breeding and Diet. Afr J Mar Sci 25∶475–486. 44. Crawford RJM, Cooper J, Dyer BM, Greyling MD, Klages NTW, Nel DC, Nel JL, Petersen SL, Wolfaardt AC (2003) Decrease in Numbers of the Eastern Rockhopper Penguin *Eudyptes chrysocome filholi* at Marion Island, 1994/95–2002/03. Afr J Mar Sci 25∶487–498. (DOC)

Table S2 Summary of the MADIFA model for wintering *Eudyptes chrysolophus* from Crozet and Kerguelen Islands. Values indicate % of variance explained by the three first principal components of the PCAs and scores of the variables on those components. Abbreviations used for the variables: BATHY: bathymetry, BATHYG: gradient of bathymetry, SST: sea-surface temperature, SSTG: gradient of SST, SSTA: SST anomaly, MLD: mixed-layer depth, CHLA: sea-surface chlorophyll *a* concentration, EKE: eddy kinetic energy. (DOC)

Table S3 Summary of the MADIFA model for wintering *Eudyptes filholi* from Crozet and Kerguelen. Values indicate % of variance explained by the three first principal components of the PCAs and scores of the variables on those components. Abbreviations used for the variables: BATHY: bathymetry, BATHYG: gradient of bathymetry, SST: sea-surface temperature, SSTG: gradient of SST, SSTA: SST anomaly, MLD: mixed-layer depth, CHLA: sea-surface chlorophyll *a* concentration, EKE: eddy kinetic energy. (DOC)

## References

[1] Alerstam T, Hedenstrom A, Akesson S (2003) Long-distance migration: evolution and determinants. Oikos 103: 247–260.

[2] Dingle H, Drake VA (2007) What is migration? Bioscience 57: 113–121.

[3] Thiebot JB, Lescroël A, Pinaud D, Trathan PN, Bost CA (2011) Larger foraging range but similar habitat selection in non-breeding versus breeding sub-Antarctic penguins. Antarct Sci 23: 117–126.

[4] Mueller T, Fagan WF (2008) Search and navigation in dynamic environments - from individual behaviors to population distributions. Oikos 117: 654–664.

[5] Guilford T, Freeman R, Boyle D, Dean B, Kirk H, et al (2011) A dispersive migration in the Atlantic Puffin and its implications for migratory navigation. PLoS ONE 6: e21336. 21799734 10.1371/journal.pone.0021336 PMC3140476

[6] Weimerskirch H (2007) Are seabirds foraging for unpredictable resources? Deep-Sea Res Part I 54: 211–223.

[7] Phillips RA, Silk JRD, Croxall JP, Afanasyev V, Bennett VJ (2005) Summer distribution and migration of nonbreeding albatrosses: Individual consistencies and implications for conservation. Ecology.

[8] Thiebot JB, Cherel Y, Trathan PN, Bost CA (2011) Inter-population segregation in the wintering areas of macaroni penguins. Mar Ecol Prog Ser 421: 279–290.

[9] Ballard G, Toniolo V, Ainley DG, Parkinson CL, Arrigo KR, et al (2010) Responding to climate change: Adélie Penguins confront astronomical and ocean boundaries. Ecology 91: 2056–2069. 20715628 10.1890/09-0688.1

[10] González-Solís J, Felicísimo A, Fox JW, Afanasyev V, Kolbeinsson Y, et al (2009) Influence of sea surface winds on shearwater migration detours. Mar Ecol Prog Ser 391: 221–230.

[11] Pinet P, Jaeger A, Cordier E, Potin G, Le Corre M (2011) Celestial moderation of tropical seabird behavior. PLoS ONE 6: e27663. 22110711 10.1371/journal.pone.0027663 PMC3215727

[12] Wilson RP, Culik B, Kosiorik P, Adelung D (1998) The overwinter movements of a chinstrap penguin. Polar Rec 34: 107–112.

[13] Bost CA, Thiebot JB, Pinaud D, Cherel Y, Trathan PN (2009) Where do penguins go during the interbreeding period? Using geolocation to track the winter dispersion of the macaroni penguin. Biol Lett 5: 473–476. 19447814 10.1098/rsbl.2009.0265 PMC2781933

[14] Wilson RP, Kreye JA, Lucke K, Urquhart H (2004) Antennae on transmitters on penguins: balancing energy budgets on the high wire. J Exp Biol 207: 2649–2662. 15201297 10.1242/jeb.01067

[15] Williams TD (1995) The Penguins. Oxford: Oxford University Press. 295 p.

[16] Warham J (1975) The Crested Penguins. In: Stonehouse B, editor. The biology of penguins. London: Macmillan. 189–269.

[17] Pütz K, Raya Rey A, Schiavini A, Clausen AP, Lüthi BH (2006) Winter migration of rockhopper penguins (*Eudyptes c. chrysocome*) breeding in the Southwest Atlantic: is utilisation of different foraging areas reflected in opposing population trends? Polar Biol 29: 735–744.

[18] Thiebot JB, Cherel Y, Trathan PN, Bost CA (2012) Coexistence of oceanic predators on wintering areas explained by population-scale foraging segregation in space or time. Ecology 93: 122–130. 22486093 10.1890/11-0385.1

[19] Cairns DK (1989) The regulation of seabird colony size – a Hinterland model. Am Nat 134: 141–146.

[20] Cherel Y, Charrassin JB, Challet E (1994) Energy and protein requirements for molt in the king penguin *Aptenodytes patagonicus* . Am J Physiol 266 R1182–R1188: 2386–2396. 10.1152/ajpregu.1994.266.4.R1182 8184961

[21] Green JA, Boyd IL, Woakes AJ, Warren NL, Butler PJ (2009) Evaluating the prudence of parents: daily energy expenditure throughout the annual cycle of a free-ranging bird, the macaroni penguin *Eudyptes chrysolophus* . J Avian Biol 40: 529–538.

[22] Sutherland WJ (1998) Evidence for flexibility and constraint in migration systems. J Avian Biol 29: 441–446.

[23] Grémillet D, Dell’Omo G, Ryan PG, Peters G, Ropert-Coudert Y, et al (2004) Offshore diplomacy, or how seabirds mitigate intra-specific competition: a case study based on GPS tracking of Cape gannets from neighbouring colonies. Mar Ecol Prog Ser 268: 265–279.

[24] Trathan PN, Green C, Tanton J, Peat H, Poncet J, et al (2006) Foraging dynamics of macaroni penguins *Eudyptes chryolophus* at South Georgia during brood-guard. Mar Ecol Prog Ser 323: 239–251.

[25] Ward P, Zahavi A (1973) Importance of certain assemblages of birds as information-centers for food-finding. Ibis 115: 517–534.

[26] Clark CW, Mangel M (1984) Foraging and flocking strategies – Information in an uncertain environment. Am Nat 123: 626–641.

[27] Weimerskirch H, Bertrand S, Silva J, Marques JC, Goya E (2010) Use of social information in seabirds: compass rafts indicate the heading of food patches. PLoS ONE 5: 9928–9936. 10.1371/journal.pone.0009928 PMC2847911 20360959

[28] González-Solís J, Croxall JP, Oro D, Ruiz X (2007) Trans-equatorial migration and mixing in the wintering areas of a pelagic seabird. Front Ecol Environ 5: 297–301.

[29] Belkin IM, Gordon AL (1996) Southern Ocean fronts from the Greenwich meridian to Tasmania. J Geophys Res C 101: 3675–3696.

[30] McDougall I, Verwoerd WJ, Chevallier L (2001) K–Ar geochronology of Marion Island, Southern Ocean. Geol Mag 138: 1–17.

[31] Giret A, Weis D, Zhou X, Cottin JY, Tourpin S (2003) Géologie des îles Crozet. Géologues 137: 15–23.

[32] Giret A, Weis D, Grégoire M, Mattielli N, Moine B, et al (2003) L’Archipel de Kerguelen: les plus vieilles îles dans le plus jeune océan. Géologues 137: 23–40.

[33] Nougier J (1982) Volcanism of Saint Paul and Amsterdam Islands (TAAF); some aspects of volcanism along plate margins. In: Craddock C, editor. Antarctic Geoscience. Madison: University of Wisconsin Press. 755–765.

[34] Baker AJ, Pereira SL, Haddrath OP, Edge KA (2006) Multiple gene evidence for expansion of extant penguins out of Antarctica due to global cooling. Proc R Soc B 273: 11–17. 10.1098/rspb.2005.3260 PMC1560011 16519228

[35] Clarke JA, Ksepka DT, Stucchie M, Urbina M, Giannini N, et al (2007) Paleogene equatorial penguins challenge the proposed relationship between biogeography, diversity, and Cenozoic climate change. Proc Natl Acad Sci USA 104: 11545–11550. 17601778 10.1073/pnas.0611099104 PMC1913862

[36] de Dinechin M, Ottvall R, Quillfeldt P, Jouventin P (2009) Speciation chronology of rockhopper penguins inferred from molecular, geological and palaeoceanographic data. J Biogeogr 36: 693–702.

[37] Gill B, Martinson P (1991) New Zealand’s Extinct Birds. Auckland: Random Century. 109 p.

[38] Brooke MD (2004) The food consumption of the world’s seabirds. Proc R Soc B 271: S246–S248. 10.1098/rsbl.2003.0153 PMC1810044 15252997

[39] Crossin GT, Trathan PN, Crawford RJM (2013) The macaroni penguin and royal penguin. In: Garcia-Borboroglu P, Boersma PD, editors. Penguins Natural history and conservation. Washington: University of Washington Press. In press.

[40] Banks J, Van Buren A, Cherel Y, Whitfield JB (2006) Genetic evidence for three species of rockhopper penguins, *Eudyptes chrysocome* . Polar Biol 30: 61–67.

[41] Stahl JC, Derenne P, Jouventin P, Mougin JL, Teulières L, et al (1985) Le cycle reproducteur des gorfous de l’archipel Crozet: *Eudyptes chrysolophus*, le Gorfou macaroni, et *Eudyptes chrysocome*, le Gorfou sauteur. Oiseau Rev Fr Ornithol 55: 27–43.

[42] Duroselle T, Tollu B (1977) The Rockhopper Penguin (*Eudyptes chrysocome moseleyi*) of Saint Paul and Amsterdam Islands. In: Llano GA, editor. Adaptations within Antarctic Ecosystems: Proceedings of the Third SCAR Symposium on Antarctic Biology. Washington: Smithsonian Institute. 579–604.

[43] Crawford RJM, Cooper J, Dyer BM (2003) Population of the Macaroni Penguin *Eudyptes chrysolophus* at Marion Island, 1994/95–2002/03, with Information on Breeding and Diet. Afr J Mar Sci 25: 475–486.

[44] Crawford RJM, Cooper J, Dyer BM, Greyling MD, Klages NTW, et al (2003) Decrease in Numbers of the Eastern Rockhopper Penguin *Eudyptes chrysocome filholi* at Marion Island, 1994/95–2002/03. Afr J Mar Sci 25: 487–498.

[45] Argos User’s Manual (2011) Worldwide tracking and environmental monitoring by satellite. Toulouse: CLS. 62 p.

[46] Wilson RP, Ducamp JJ, Rees G, Culik BM, Niekamp K (1992) Estimation of location: global coverage using light intensity. In: Priede IM, Swift SM, editors. Wildlife telemetry: remote monitoring and tracking of animals. Chichester: Ellis Howard. 131–134.

[47] Hill RD (1994) Theory of geolocation by light levels. In: Le Boeuf BJ, Laws RM, editors. Elephant seals: population ecology, behaviour and physiology. Berkeley: University of California Press. 227–236.

[48] Thiebot JB, Pinaud D (2010) Quantitative method to estimate species habitat use from light-based geolocation data. Endang Species Res 10: 341–353.

[49] R Development Core Team (2009) R: a language and environment for statistical computing. R Foundation for Statistical Computing, Vienna, Austria. URL http://www.R-project.org.

[50] Raya Rey A, Trathan PN, Pütz K, Schiavini A (2007) Effect of oceanographic conditions on the winter movements of rockhopper penguins *Eudyptes chrysocome chrysocome* from Staten Island, Argentina. Mar Ecol Prog Ser 330: 285–295.

[51] Wilson RP, Grémillet D, Syder J, Kierspel MAM, Garthe S, et al (2002) Remote-sensing systems and seabirds: their use, abuse and potential for measuring marine environmental variables. Mar Ecol Prog Ser 228: 241–261.

[52] Staniland IJ, Robinson SL, Silk JRD, Warren N, Trathan PN (2012) Winter distribution and haul-out behaviour of female Antarctic fur seals from South Georgia. Mar Biol 159: 291–301.

[53] Bost CA, Charrassin JB, Clerquin Y, Ropert-Coudert Y, Le Maho Y (2004) Exploitation of distant marginal ice zones by king penguins during winter. Mar Ecol Prog Ser 283: 293–297.

[54] Freitas C, Lydersen C, Fedak MA, Kovacs KM (2008) A simple new algorithm to filter marine mammal Argos locations. Mar Mamm Sci 24: 315–325.

[55] Brown CR (1987) Traveling speed and foraging range of macaroni and rockhopper penguins at Marion Island. J Field Ornithol 58: 118–125.

[56] Calenge C, Darmon G, Basille M, Loison A, Jullien JM (2008) The factorial decomposition of the Mahalanobis distances in habitat selection studies. Ecology 89: 555–566. 18409444 10.1890/06-1750.1

[57] Tsoar A, Allouche O, Steinitz O, Rotem D, Kadmon R (2007) A comparative evaluation of presence-only methods for modelling species distribution. Diversity Distrib 13: 397–405.

[58] Cotté C, Park YH, Guinet C, Bost CA (2007) Movements of foraging king penguins through marine mesoscale eddies. Proc R Soc B 274: 2385–2391. 10.1098/rspb.2007.0775 PMC2274980 17669726

[59] Bost CA, Goarant A, Scheffer A, Koubbi P, Duhamel G, et al. (2011) Foraging habitat and performances of King penguins *Aptenodytes patagonicus*, Miller, 1778 at Kerguelen islands in relation to climatic variability. In: Duhamel G, Welsford D, editors. The Kerguelen Plateau: Marine Ecosystem and Fisheries. Paris: Société Française d’Ichtyologie. 199–202.

[60] Péron C, Weimerskirch H, Bost CA (2012) Projected poleward shift of king penguins’ (*Aptenodytes patagonicus*) foraging range at the Crozet Islands, southern Indian Ocean. Proc R Soc B 279: 2515–2523. 10.1098/rspb.2011.2705 PMC3350699 22378808

[61] Clarke A (1988) Seasonality in the Antarctic marine environment. Comp. Biochem. Physiol. B 90: 461–473.

[62] Cherel Y, Hobson KA, Guinet C, Vanpe C (2007) Stable isotopes document seasonal changes in trophic niches and winter foraging individual specialization in diving predators from the Southern Ocean. J Anim Ecol 76: 826–836. 17584388 10.1111/j.1365-2656.2007.01238.x

[63] Trivelpiece WZ, Buckelew S, Reiss C, Trivelpiece SG (2007) The winter distribution of chinstrap penguins from two breeding sites in the South Shetland Islands of Antarctica. Polar Biol 30: 1231–1237.

[64] Stahl JC, Bartle JA, Jouventin P, Roux JP, Weimerskirch H (1996) Atlas of seabird distribution in the south-west Indian ocean. Villiers-en-Bois: Centre National de la Recherche Scientifique. 226 p.

[65] Tremblay Y, Cherel Y (1999) Synchronous underwater foraging behavior in penguins. Condor 101: 179–185.

[66] Boinski S, Garber PA (2000) On the move, how and why animals travel in groups. Chicago: Chicago University Press. 822 p.

[67] Friesen VL, Burg TM, McCoy KD (2007) Mechanisms of population differentiation in seabirds. Mol Ecol 16: 1765–1785. 17444891 10.1111/j.1365-294X.2006.03197.x

[68] Mayr E (1963) Animal species and evolution. Cambridge: Harvard University Press. 797 p.

[69] Hull CL (1999) The foraging zones of breeding royal (*Eudyptes schlegeli*) and rockhopper (*E. chrysocome*) penguins: an assessment of techniques and species comparison. Wildl Res 26: 789–803.

[70] Hull CL (2000) Comparative diving behaviour and segregation of the marine habitat by breeding Royal Penguins, *Eudyptes schlegeli*, and eastern Rockhopper Penguins, *Eudyptes chrysocome filholi*, at Macquarie Island. Can J Zool 78: 333–345.

[71] Ridoux V (1994) The diets and dietary segregation of seabirds at the subantarctic Crozet Islands. Mar Ornithol 22: 1–192.

[72] Hull CL (1999) Comparison of the diets of breeding royal (*Eudyptes schlegeli*) and rockhopper (*Eudyptes chrysocome*) penguins on Macquarie Island over three years. J Zool Lond 247: 507–529.

[73] Wilson RP (1995) Foraging Ecology. In: Perrins CM, Bock WJ, Kikkawa J, editors. The Penguins. Oxford: Oxford University Press. 81–106.

[74] Wilson RP (2010) Resource partitioning and niche hyper-volume overlap in free-living Pygoscelid penguins. Func Ecol 24: 646–657.

[75] Andersen R (1991) Habitat deterioration and the migratory behavior of moose (*Alces alces* L) in Norway. J Appl Ecol 28: 102–108.

[76] Berthold P, Helbig AJ, Mohr G, Querner U (1992) Rapid microevolution of migratory behavior in a wild bird species. Nature 360: 668–670.

[77] Berthold P (1999) A comprehensive theory for the evolution, control and adaptability of avian migration. Ostrich 70: 1–11.

[78] Hamer KC, Schreiber EA, Burger J (2002) Breeding biology, life histories, and life history-environment interactions in seabirds. In: Schreiber EA, Burger J, editors. Biology of Marine Birds. Boca Raton: CRC Press. 217–261.

[79] Becquey S, Gersonde R (2002) Past hydrographic and climatic changes in the Subantarctic Zone of the South Atlantic - The Pleistocene record from ODP Site 1090. Palaeogeogr Palaeoclimatol Palaeoecol 182: 221–239.

[80] Kemp AES, Grigorov I, Pearce RB, Naveira Garabato AC (2010) Migration of the Antarctic Polar Front through the mid-Pleistocene transition: evidence and climatic implications. Quat Sci Rev 29: 1993–2009.

[81] Cresswell KA, Wiedenmann J, Mangel M (2008) Can macaroni penguins keep up with climate- and fishing-induced changes in krill? Polar Biol 31: 641–649.

[82] Frederiksen M, Moe B, Daunt F, Phillips RA, Barrett RT, et al (2012) Multicolony tracking reveals the winter distribution of a pelagic seabird on an ocean basin scale. Diversity Distrib 18: 530–542.

[83] IUCN (2012) IUCN Red List of Threatened Species. Version 2012.2. Available: http://www.iucnredlist.org. Accessed 05 November 2012.

[84] Robson B, Glass T, Glass N, Glass J, Green J, et al (2011) Revised population estimate and trends for the Endangered Northern Rockhopper Penguin *Eudyptes moseleyi* at Tristan da Cunha. Bird Conserv Int 21: 454–459.

