# Supplementary figures and images for: A Space Oddity: Geographic and Specific Modulation of Migration in Eudyptes Penguins
Source: PLoS One. 2013 Aug 2;8(8):e71429. doi: 10.1371/journal.pone.0071429 (PMC3732226; doi:10.1371/journal.pone.0071429)

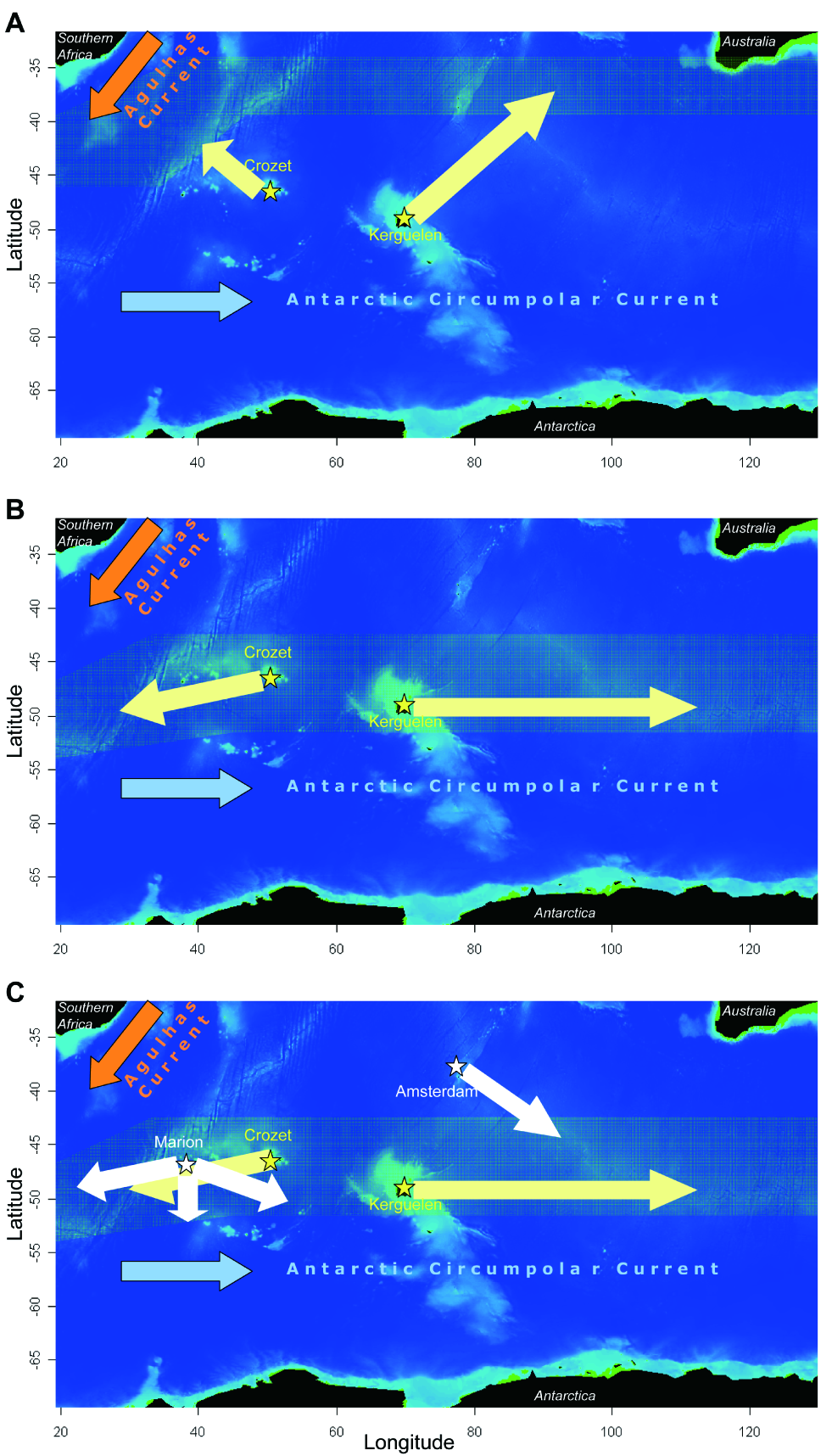

Supplement: Figure S1 — (TIF) [file pone.0071429.s001.tif]
