# Supplementary material for: A Space Oddity: Geographic and Specific Modulation of Migration in Eudyptes Penguins
Source: PLoS One. 2013 Aug 2;8(8):e71429. doi: 10.1371/journal.pone.0071429 (PMC3732226; doi:10.1371/journal.pone.0071429)
Supplement: Table S1 — (DOC) [file pone.0071429.s002.doc]

**Table S1.**

| **Species** | **Locality** | **Departure dates** | **Return dates** | **Tagging period** |
| --- | --- | --- | --- | --- |
| *E. chrysolophus* | Marion | 14 Apr. ^*^ | 24 Oct. ^[41]^ | 1 – 16 Apr. |
| *E. chrysolophus* | Crozet | 30 Apr. ^[8]^ | 31 Oct. ^[8]^ | 27 – 29 Apr. |
| *E. chrysolophus* | Kerguelen | 19 Apr. ^[13]^ | 24 Oct. ^[13]^ | 24 Mar. – 17 Apr. |
| *E. filholi* | Marion | 5 May * | 9 Nov. ^[42]^ | 16 Apr. – 3 May |
| *E. filholi* | Crozet | 13 May ^[18]^ | 8 Nov. ^[18]^ | 9 – 11 May |
| *E. filholi* | Kerguelen | 11 May ^[18]^ | 25 Nov. ^[18]^ | 5 – 8 May |
| *E. moseleyi* | Amsterdam | 16 Mar. ^[18]^ | 25 Jul. ^[18]^ | 22 Feb. – 1 Mar. |
