# Supplementary material for: A Space Oddity: Geographic and Specific Modulation of Migration in Eudyptes Penguins
Source: PLoS One. 2013 Aug 2;8(8):e71429. doi: 10.1371/journal.pone.0071429 (PMC3732226; doi:10.1371/journal.pone.0071429)
Supplement: Table S2 — (DOC) [file pone.0071429.s003.doc]

**Table S2.**

| **First PCA** | **Component 1** | **Component 2** | **Component 3** |
| --- | --- | --- | --- |
| % of variance | 29.7 | 21.1 | 15.4 |
| BATHY | -0.11 | 0.43 | -0.52 |
| BATHYG | 0.03 | -0.58 | 0.33 |
| SST | -0.58 | 0.20 | 0.15 |
| SSTG | 0.40 | -0.41 | -0.40 |
| SSTA | -0.28 | -0.17 | -0.19 |
| MLD | 0.43 | 0.25 | -0.04 |
| CHLA | -0.45 | -0.32 | -0.08 |
| EKE | -0.16 | -0.27 | -0.63 |
| **Second PCA** | **Component 1** | **Component 2** | **Component 3** |
| % of variance | 79.0 | 10.9 | 5.0 |
| BATHY | 0.23 | 0.14 | -0.02 |
| BATHYG | -0.22 | 0.20 | 0.01 |
| SST | 0.95 | 0.42 | -0.09 |
| SSTG | -0.73 | -0.01 | 0.20 |
| SSTA | 0.18 | 0.48 | 0.02 |
| MLD | -0.16 | -0.85 | 0.01 |
| CHLA | 0.32 | 0.73 | 0.33 |
| EKE | 0.14 | 0.12 | 0.96 |
