# Supplementary material for: A Space Oddity: Geographic and Specific Modulation of Migration in Eudyptes Penguins
Source: PLoS One. 2013 Aug 2;8(8):e71429. doi: 10.1371/journal.pone.0071429 (PMC3732226; doi:10.1371/journal.pone.0071429)
Supplement: Table S3 — (DOC) [file pone.0071429.s004.doc]

**Table S3.**

| **First PCA** | **Component 1** | **Component 2** | **Component 3** |
| --- | --- | --- | --- |
| % of variance | 25.7 | 19.8 | 16.0 |
| BATHY | 0.34 | 0.62 | 0.05 |
| BATHYG | -0.38 | -0.56 | 0.23 |
| SST | -0.48 | 0.33 | -0.32 |
| SSTG | 0.02 | 0.11 | -0.12 |
| SSTA | 0.15 | 0.07 | 0.73 |
| MLD | 0.31 | -0.27 | -0.53 |
| CHLA | -0.56 | 0.24 | -0.00 |
| EKE | -0.29 | 0.20 | 0.10 |
| **Second PCA** | **Component 1** | **Component 2** | **Component 3** |
| % of variance | 99.5 | 0.26 | 0.18 |
| BATHY | -0.06 | -0.13 | -0.24 |
| BATHYG | 0.04 | -0.03 | -0.11 |
| SST | -0.01 | -0.88 | 0.54 |
| SSTG | -0.99 | -0.01 | 0.02 |
| SSTA | 0.01 | 0.19 | -0.40 |
| MLD | -0.03 | 0.58 | 0.72 |
| CHLA | 0.00 | -0.65 | 0.14 |
| EKE | 0.01 | -0.13 | -0.01 |
